# Frailty measurement and its contribution to clinical care and health services: a commentary

**DOI:** 10.1186/s13584-018-0225-0

**Published:** 2018-05-23

**Authors:** Shannon Wu, Bruce Leff

**Affiliations:** 1 Department of Health Policy and Management, Johns Hopkins Bloomberg School of Public Health, Baltimore, MD 21205 USA; 2 Division of Geriatric Medicine and Gerontology, Center for Transformative Geriatric Research, Johns Hopkins University School of Medicine, Baltimore, MD 21224 USA

**Keywords:** Frailty, Measurement, Health services

## Abstract

Frailty is associated with poorer quality of life and higher healthcare utilization and spending. Despite its importance, no clear consensus exists on the definition of frailty. The recent IJHPR article by Buch et al. significantly contributes to the advancement of Israel’s understanding of frailty by estimating for the first time the prevalence of frailty in the country. This commentary discusses the context of past and current advancements in measuring frailty and discusses how frailty measurement can contribute to both clinical care and the organization of health services to care for frail older adults in Israel and other developed countries.

## Background

The important IJHPR paper by Buch and colleagues characterizes, for the first time, frailty prevalence in Israel’s older population [1]. The Central Bureau of Statistics estimates that Israel’s elderly population will double to 1.64 million in 2035, one of the highest growth rates in the over-65 category in the Western world [2]. Frailty, both a clinical and physical manifestation of old age, is associated with poorer quality of life and higher healthcare utilization and spending [3]. As the authors state, information on the prevalence of frailty may be helpful in efforts “to minimize its occurrence and constrain its social, economic, and health costs in the face of a rapid rise in the elderly population.” We place Buch’s paper in the context of past and current advancements in measuring frailty and discuss how frailty measurement can contribute to both clinical care and the organization of health services to care for frail older adults.

## Measuring frailty

Despite its importance, no clear consensus exists on the definition of frailty for clinicians, researchers, and policy makers. However, an operational definition of frailty is important for clinical care, research, and policy planning [4]. Clinicians need to screen and care for their patients; researchers aim to explore the etiology and predictors of frailty and evaluate interventions to attenuate or reverse its progress; health care delivery organizations need to develop and implement care models to best care for the frail; and policy makers need to decide on the appropriate allocation of resources and future health system planning for an aging population.

Two main theories have dominated the field of frailty measurement. Linda Fried and colleagues characterize frailty as a phenotype consisting of weight loss, weakness, exhaustion, low activity level, and slow gait speed [5]. This construct is one that mostly focuses on physical frailty, a “wasting disorder” that is distinguished from comorbidity and from disability. Researchers have drawn from this model for investigations of the biology of frailty. In contrast, an “accumulation of deficits” model, developed by Kenneth Rockwood and colleagues, counts the number of comorbidities and disabilities that can accumulate to frailty [6]. This model is based on the premise that as an individual has more things wrong, no matter that they are, the more frail the individual will be. The accumulation of these clinical manifestations leads to increased vulnerability and suggests that no system exists in isolation. The two concepts of frailty by Fried and Rockwood have advanced the field in operationalizing this complex manifestation.

Other instruments and approaches to assess for frailty exist. It is not easy to operationalize frailty nor is it easy to compare definitions empirically because different definitions serve different purposes. Sternberg et al. [4] and Buta et al. [7] provide useful systematic reviews of highly-cited instruments. From these various methods, we see two contentious themes emerge: 1) defining what components to include to identify frailty; and 2) determining whether these components act as risk factors for frailty or as outcomes of frailty. For example, components of physical function and activities of daily living are used both as criteria of frailty and as outcomes of frailty. As Buch and colleagues astutely point out, “given the variety of diagnostic approaches, it is hardly surprising that the estimated prevalence of frailty in the elderly ranges between 5 and 58%.” Thus, the choice of these components has implications for the type of tool created to screen and to intervene with clinical and other care management practices. One approach is to measure clinical attributes that suggest frailty, broadly writ, so that interventions can be introduced earlier in their care.

Recent advancements in the measurement of frailty have focused on the relationship of cognitive, social, and mood risk factors, and on its dynamic process rather than considering it as a static condition. For example, social vulnerability and psychological factors have been demonstrated to be correlated with frailty [8]; and together frailty and cognitive factors lead to acceleration in age-related declines [9]. These developments have led to considering frailty as a geriatric syndrome. This conceptualization of frailty captures the multifactorial conditions and their interactions that lead to a unified manifestation [10]. The implications of this dynamic and multifactorial approach are two-fold. First, clinicians and researchers should examine complex and shared etiological pathways and test unified intervention strategies on shared risk factors to reduce poor outcomes later in life. Second, the ability to translate these complex strategies into evidence-based practice and policy would require coordinated and multifaceted approaches.

Buch et al. begin to address these issues in the Israeli population by examining frailty prevalence and exploring a screening tool within a nationally representative data. They anchor their definition of frailty to both Fried’s and Rockwood’s conceptualizations, combining a phenotype and accumulation of deficit outlook. It is important to highlight here that the five components used in their definition are physical inactivity, number of comorbidities, weight loss, sarcopenia, and low subjective health perception (for which we applaud the inclusion of a patient psychological measure). They find that 5% of the Israeli population aged 65 and older can be qualified as frail and 57% as pre-frail. The authors also find significant differences by gender, education, and income levels, with females, less educated, and low-income groups more likely to be frail than non-frail. Given these findings, it will be important to organize healthcare and service delivery to plan and prioritize resources for these high-risk groups in an increasingly aging Israeli population.

## Using frailty measurement to advance health care delivery for older adults

In the context of health service delivery, the ultimate goals of measuring frailty and factors associated with frailty are to 1) identify frail individuals for clinical care and care management; 2) develop and implement interventions to attenuate and/or prevent decline earlier in the frailty development trajectory; and 3) establish organizational strategies and services that can optimize care for frail older adults. In the global context, a rapidly aging population necessitates the practical understanding of such approaches and the development of coordinated interventions to improve the quality of life for frail older adults.

Data advances are promising for using existing administrative billing data and/or primary care electronic health record data to create indices to identify frail patients [11–13]. Traditional assessments, such as the comprehensive geriatric assessment, are time consuming and expensive for providers, health systems, and policy makers to conduct on a large scale. They also lack information on the dynamic process that occurs in frailty development. Large scale screening tools at the point of care may be feasible as developments in data infrastructure continue to advance. These may be especially helpful in identifying patients most likely to benefit from innovative clinical models that care for frail older adults with and without other behavioral and social needs, and in identifying those most likely to benefit from underutilized approaches such as physical therapy, social services, or polypharmacy reduction. Examples of these models include Improving Mood-Promoting Access to Collaborative Treatment (IMPACT) [14], Community Aging in Place, Advancing Better Living for Elders (CAPABLE) [15], Maximizing Independence (MIND) at Home [16], home-based primary care [17], home-based palliative care [18], and hospital at home [19]. These specifically designed programs may positively affect individual, caregiver, and system outcomes.

Health systems and policy have also begun to move toward an explicit acknowledgment of the need for caring for frail patients in organizational and payment policies. In the United States, the Program of All-Inclusive Care for the Elderly (PACE) has long held the tradition of incorporating explicit frailty measurement, beyond traditional comorbidity in older adults, as a method to ensure patient access to and financial fairness for providers who care for a higher proportion of frail older adults [20]. In the UK, the National Health Service recently recognized the need for frailty identification among general practices. In the GP 2017/18 contract, general practices are required to identify all patients aged 65 and over who may be living with moderate or severe frailty to target those most at risk for adverse events [21].

## Conclusion

Israel, in its first step in surveying the prevalence of frailty in its elderly population, is moving forward in creating a health system that is more sensitive in caring for an aging population. While doing so, it will be important to keep in context the implications and goals of various measurement methods on strategies and policies aimed at providing this care.
